# Importance of Rare *DPYD* Genetic Polymorphisms for 5-Fluorouracil Therapy in the Japanese Population

**DOI:** 10.3389/fphar.2022.930470

**Published:** 2022-06-15

**Authors:** Eiji Hishinuma, Yoko Narita, Kai Obuchi, Akiko Ueda, Sakae Saito, Shu Tadaka, Kengo Kinoshita, Masamitsu Maekawa, Nariyasu Mano, Noriyasu Hirasawa, Masahiro Hiratsuka

**Affiliations:** ^1^ Advanced Research Center for Innovations in Next-Generation Medicine, Tohoku University, Sendai, Japan; ^2^ Tohoku Medical Megabank Organization, Tohoku University, Sendai, Japan; ^3^ Laboratory of Pharmacotherapy of Life-Style Related Diseases, Graduate School of Pharmaceutical Sciences, Tohoku University, Sendai, Japan; ^4^ Graduate School of Information Sciences, Tohoku University, Sendai, Japan; ^5^ Department of Pharmaceutical Sciences, Tohoku University Hospital, Sendai, Japan

**Keywords:** dihydropyrimidine dehydrogenase (DPD), DPYD, 5-fluorouracil, pharmacogenomics, genetic polymorphism

## Abstract

Dihydropyrimidine dehydrogenase (DPD), encoded by the *DPYD* gene, is the rate-limiting enzyme in 5-fluorouracil (5-FU) degradation. In Caucasians, four *DPYD* risk variants are recognized to be responsible for interindividual variations in the development of 5-FU toxicity. However, these risk variants have not been identified in Asian populations. Recently, 41 *DPYD* allelic variants, including 15 novel single nucleotide variants, were identified in 3,554 Japanese individuals by analyzing their whole-genome sequences; however, the effects of these variants on DPD enzymatic activity remain unknown. In the present study, an *in vitro* analysis was performed on 41 DPD allelic variants and three DPD risk variants to elucidate the changes in enzymatic activity. Wild-type and 44 DPD-variant proteins were heterologously expressed in 293FT cells. DPD expression levels and dimerization of DPD were determined by immunoblotting after SDS-PAGE and blue native PAGE, respectively. The enzymatic activity of DPD was evaluated by quantification of dihydro-5-FU, a metabolite of 5-FU, using high-performance liquid chromatography-tandem mass spectrometry. Moreover, we used 3D simulation modeling to analyze the effect of amino acid substitutions on the conformation of DPD. Among the 41 DPD variants, seven exhibited drastically decreased intrinsic clearance (*CL*_*int*_) compared to the wild-type protein. Moreover, R353C and G926V exhibited no enzymatic activity, and the band patterns observed in the immunoblots after blue native PAGE indicated that DPD dimerization is required for its enzymatic activity. Our data suggest that these variants may contribute to the significant inter-individual variability observed in the pharmacokinetics and pharmacodynamics of 5-FU. In our study, nine DPD variants exhibited drastically decreased or no enzymatic activity due to dimerization inhibition or conformational changes in each domain. Especially, the rare *DPYD* variants, although at very low frequencies, may serve as important pharmacogenomic markers associated with the severe 5-FU toxicity in Japanese population.

## Introduction

**FIGURE 1:**
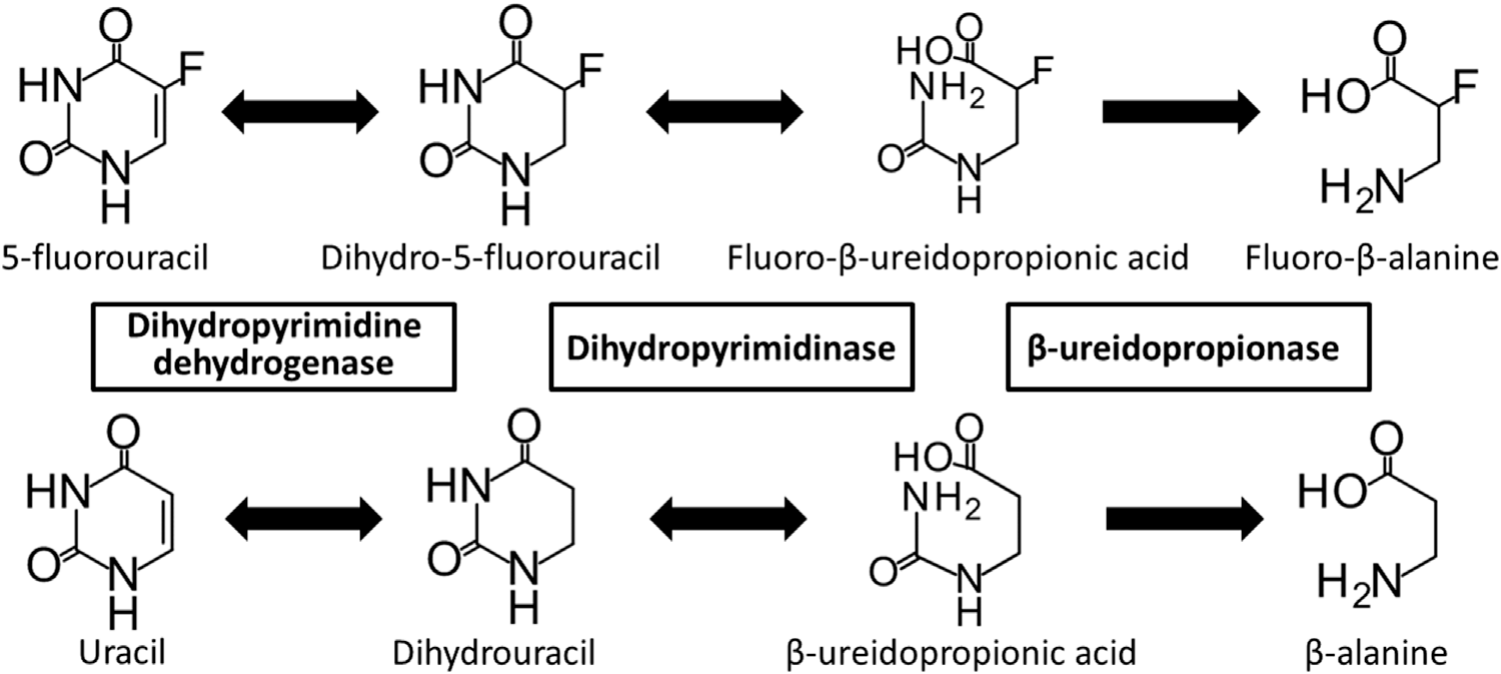
Metabolic pathway of 5-fluorouracil and uracil. 5-Fluorouracil and uracil are catabolized by dihydropyrimidine dehydrogenase, dihydropyrimidinase, and β-ureidopropionase.

Dihydropyrimidine dehydrogenase (DPD; EC 1.3.1.2) is a rate-limiting enzyme involved in the degradation of the endogenous nucleobases uracil and thymine, and the anticancer drug 5-fluorouracil (5-FU) (Kikugawa et al., 1994; Gmeiner, 2021). 5-FU is phosphorylated and activated intracellularly, causing inhibition of DNA synthesis and RNA dysfunction (Wigmore et al., 2010). Although 5-FU is a key drug used to treat many solid tumors, including gastric, colorectal, and breast cancers, it causes severe side effects, such as neutropenia, thrombocytopenia, mucositis, diarrhea, and hand-foot syndrome, in 10%–30% of patients treated with 5-FU (Wigmore et al., 2010; Kobuchi and Ito, 2020). More than 80% of administered 5-FU is excreted through a three-step metabolic pathway involving DPD (Figure 1) (Heggie et al., 1987; Kunicka et al., 2016). First, DPD catalyzes the reduction of 5-FU to dihydro-5-FU (FUH_2_). Dihydropyrimidinase (DHP; EC 3.5.2.2) then catalyzes the hydrolysis of FUH_2_. Finally, the fluoro-β-ureidopropionic acid (FUPA) produced as a result of the previous steps is converted to fluoro-β-alanine by β-ureidopropionase (β-UP, EC 3.5.1.6). A loss of function of any of these enzymes can cause severe 5-FU toxicity; in particular, the loss of function of DPD, the rate-limiting enzyme, is an important factor (Botticelli et al., 2016; Vodenkova et al., 2020). DPD deficiency may result in increased anti-tumor effects or toxicity due to increased blood levels and accumulation of 5-FU (Beck et al., 1994; Botticelli et al., 2016).

DPD is expressed in almost all cells, but is particularly active in the liver and lymphocytes (van Kuilenburg et al., 2006). DPD is encoded by the *DPYD* gene, which is located on chromosome 1p21, comprises 23 exons, and consists of a 3,078 bp open-reading frame that encodes a polypeptide containing 1,025 amino acid residues (Lu et al., 1992). Single nucleotide variants (SNV), deletions, and insertions in *DPYD* can lead to changes in the amino acid sequence of DPD that alter its enzymatic activity. In addition, microdeletion and chromosomal instability in the 1p21 region of *DPYD* may cause DPD deficiency (Brečević et al., 2015), which is an autosomal recessive genetic disorder with clinical symptoms that vary widely and include seizures, mental developmental disorders, microcephaly, autism, and asymptomatic individuals (van Kuilenburg, 2004; van Kuilenburg et al., 2009). It is important to be able to predict the risk of developing toxicity because a DPD deficiency may only be detected after 5-FU chemotherapy leads to severe toxicity in asymptomatic patients (Ezzeldin and Diasio, 2004).

To date, more than 450 genetic variants have been identified in the *DPYD* gene (Sistonen et al., 2012; Thomas et al., 2016; Hishinuma et al., 2020). In Caucasians, four *DPYD* risk variants, including c.1905+1G>A (IVS14+1G>A, DPYD*2A), c.1129-5923C>G/hapB3, c.1679T>G (DPYD*13, p.I560S), and c.2846A>T (p.D949V), have been reported (Froehlich et al., 2015; Meulendijks et al., 2015). These variants are known to reduce DPD enzymatic activity due to splicing defects or amino acid substitutions (Meulendijks et al., 2015; Deenen et al., 2016). The Clinical Pharmacogenetics Implementation Consortium (CPIC) and Dutch Pharmacogenetics Working Group (DPWG) have established guidelines on 5-FU dosage adjustment based on *DPYD* genetic polymorphisms (Henricks et al., 2015; Amstutz et al., 2018; Lunenburg et al., 2020). However, there are considerable geographical-ethnicity differences in the variation and frequency of *DPYD* genetic polymorphisms, and these four *DPYD* risk variants have not been identified in Asian populations, including the Japanese population (van Kuilenburg, 2004; Maekawa et al., 2007; Yokoi et al., 2020). We previously reported enzymatic functional alterations in 21 DPD variants identified by whole-genome sequencing (WGS) of 1,070 Japanese individuals, suggesting that some DPD variants specific to the Japanese population may be responsible for the development of severe 5-FU toxicity (Hishinuma et al., 2018). To accurately predict this occurrence, a comprehensive functional analysis that includes rare *DPYD* genetic polymorphisms present in the Japanese population is necessary.

Recently, 15 novel *DPYD* variants were identified by WGS of 3,554 Japanese individuals conducted by the Tohoku University Tohoku Medical Megabank Organization (ToMMo), but any functional changes resulting from these variations remain unknown (Tadaka et al., 2021). Therefore, in this study, we characterized the enzymatic activity of wild-type DPD and 41 variants derived from *DPYD* genetic polymorphisms identified by ToMMo (Tables 1, 2) using recombinant proteins expressed in 293FT cells, and determined the kinetic parameters of these variants using a 5-FU reduction activity assay. In addition, three DPD risk variants (DPYD*2A, DPYD*13, and c.2846A>T) were analyzed. Our results indicate the potential for rare *DPYD* variants to be used as novel pharmacogenomic markers for predicting severe 5-FU toxicity in the Japanese population.

**TABLE 1:** *DPYD* variants identified in 3,554 Japanese subjects and three risk variants.

db SNP rsID	Location	Nucleotide change	Amino acid substitution	Frequency (%)	References
—	Exon 2	74A > G	H25R	0.01	Hishinuma et al. (2018)
rs1801265	Exon 2	85T > C (*9A, *9B)	C29R	3.05	Offer et al. (2013)
rs527580106	Exon 3	229A > G	M77V	0.01	Novel
rs1212037891	Exon 5	325T > A	Y109N	0.23	Hishinuma et al. (2018)
rs200562975	Exon 5	451A > G	N151D	0.23	Hishinuma et al. (2018)
rs2297595	Exon 6	496A > G	M166V	2.18	Hishinuma et al. (2018)
rs371792178	Exon 6	524C > T	S175L	0.03	Hishinuma et al. (2018)
—	Exon 6	572C > T	A191V	0.01	Novel
rs767836989	Exon 7	733A > T	I245F	0.04	Novel
—	Exon 9	871G > A	D291N	0.01	Novel
rs143879757	Exon 9	893C > T	T298M	0.01	Hishinuma et al. (2018)
rs183105782	Exon 9	910T > C	Y304H	0.03	Offer et al. (2014)
—	Exon 9	937G > T	V313L	0.03	Hishinuma et al. (2018)
rs1400122790	Exon 10	963G > A	M321I	0.01	Novel
rs72549306	Exon 10	1003G > A	V335M	0.13	Hishinuma et al. (2018)
rs143154602	Exon 10	1057C > T	R353C	0.01	Offer et al. (2014)
rs1285381791	Exon 10	1061G > A	C354Y	0.03	Novel
—	Exon 10	1097G > C	G366A	0.03	Hishinuma et al. (2018)
—	Exon 11	1139C > T	A380V	0.01	Hishinuma et al. (2018)
—	Exon 11	1150A > G	K384E	0.01	Hishinuma et al. (2018)
rs748620513	Exon 11	1300G > C	V434L	0.03	Hishinuma et al. (2018)
—	Exon 11	1312T > G	F438V	0.01	Novel
rs148994843	Exon 13	1543G > A	V515I	0.03	Hishinuma et al. (2018)
rs777368221	Exon 13	1572T > G	F524L	0.06	Novel
rs1180771326	Exon 13	1582A > G	I528V	0.01	Elraiyah et al. (2017)
rs1801159	Exon 13	1627A > G (*5)	I543V	26.94	Hishinuma et al. (2018)
rs1314015254	Exon 13	1678A > G	I560V	0.03	Novel
rs55886062	Exon 13	1679T > G (*13)	I560S	—	Offer et al. (2013)
rs59086055	Exon 14	1774C > T	R592W	0.01	Hishinuma et al. (2018)
—	Exon 14	1811A > T	Q604L	0.01	Novel
rs3918290	—	1905 + 1 G > A (*2A)	Exon 14 skipping	—	Offer et al. (2013)
rs1801160	Exon 18	2194G > A (*6)	V732I	1.91	Hishinuma et al. (2018)
rs368327291	Exon 18	2212G > C	V738L	0.01	Novel
—	Exon 18	2243G > A	G748D	0.03	Novel variant
rs56005131	Exon 19	2303C > A	T768K	2.42	Hishinuma et al. (2018)
rs1335150891	Exon 19	2420A > G	H807R	0.03	Hishinuma et al. (2018)
—	Exon 20	2476G > A	V826M	0.1	Hishinuma et al. (2018)
rs571114616	Exon 20	2528T > C	I843T	0.01	Novel
rs1801267	Exon 21	2657G > A (*9B)	R886H	0.01	Offer et al. (2014)
rs188052243	Exon 21	2678A > G	N893S	0.23	Hishinuma et al. (2018)
—	Exon 22	2777G > T	G926V	0.01	Hishinuma et al. (2018)
—	Exon 22	2816G > A	S939N	0.01	Novel
rs67376798	Exon 22	2846A > T	D949V	—	Offer et al. (2014)
—	Exon 23	2969C > T	T990I	0.01	Novel

**TABLE 2:** Geographical-ethnicity differences in the variation and frequency of *DPYD* variants.

db SNP rsID	Nucleotide change	Frequency (%)	gnomAD AMR (%)	gnomAD ASJ (%)	gnomAD EAS (%)	gnomAD NFE (%)	gnomAD AFR (%)
—	74A > G	0.01					
rs1801265	85T > C (*9A, *9B)	3.05	24.74	11.07	7.23	22.54	40.32
rs527580106	229A > G	0.01					0.002
rs1212037891	325T > A	0.23			0.01		
rs200562975	451A > G	0.23	0.42		0.06	0.003	0.02
rs2297595	496A > G	2.18	6.66	8.42	1.62	10.11	3.36
rs371792178	524C > T	0.03	0.007			0.003	0.01
—	572C > T	0.01				0.001	
rs767836989	733A > T	0.04					
—	871G > A	0.01					
rs143879757	893C > T	0.01				0.007	
rs183105782	910T > C	0.03			0.04		
—	937G > T	0.03					
rs1400122790	963G > A	0.01					
rs72549306	1003G > A	0.13					0.002
rs143154602	1057C > T	0.01				0.001	
rs1285381791	1061G > A	0.03					
—	1097G > C	0.03			0.02		
—	1139C > T	0.01					
—	1150A > G	0.01					
rs748620513	1300G > C	0.03			0.04		
—	1312T > G	0.01					
rs148994843	1543G > A	0.03	0.007			0.009	
rs777368221	1572T > G	0.06			0.06		
rs1180771326	1582A > G	0.01					
rs1801159	1627A > G (*5)	26.94	23.69	19.43	25.52	19.60	15.82
rs1314015254	1678A > G	0.03					
rs55886062	1679T > G (*13)	—	0.007			0.07	0.02
rs59086055	1774C > T	0.01			0.01	0.003	0.005
—	1811A > T	0.01					
rs3918290	1905 + 1 G > A (*2A)	—	0.17	0.69		0.50	0.06
rs1801160	2194G > A (*6)	1.91	4.60	10.68	1.54	4.52	2.45
rs368327291	2212G > C	0.01					0.02
—	2243G > A	0.03			0.02		
rs56005131	2303C > A	2.42			0.50		0.01
rs1335150891	2420A > G	0.03					
—	2476G > A	0.1					
rs571114616	2528T > C	0.01					0.002
rs1801267	2657G > A (*9B)	0.01				0.001	0.007
rs188052243	2678A > G	0.23			0.04		
—	2777G > T	0.01					
—	2816G > A	0.01					
rs67376798	2846A > T	—	0.17	0.06		0.60	0.12
—	2969C > T	0.01					

Data of each frequency were referenced to gnomAD v3.1.2 (https://gnomad.broadinstitute.org/) (cited 20 May 2022). AMR, Latino/Admixed American; ASJ, ashkenazi jewish; EAS, east asian; NFE, Non-Finnish European; AFR, African/African American.

## Materials and Methods

### Chemicals and Reagents

Antibodies and reagents were purchased from the following commercial sources: cytosine-2,4-^13^C_2_,^15^N_3_ (TAIYO NIPPON SANSO, Tokyo, Japan); polyclonal anti-human DPD antibody (ABC451; Millipore, Tokyo, Japan); polyclonal anti-GAPDH (glyceraldehyde 3-phosphate dehydrogenase) antibody (G9545; Sigma-Aldrich, St. Louis, MO, United States). Other chemicals and reagents were the same as in previous reports (Hishinuma et al., 2018).

### Sanger Sequencing of *DPYD* Gene

Genomic DNA was isolated from the whole blood of Japanese participants in a cohort study conducted by ToMMo (Kuriyama et al., 2016; Minegishi et al., 2019; Nagami et al., 2020). All study participants provided informed consent, and the study was approved by the Tohoku University Graduate School of Pharmaceutical Sciences Ethics Committee (permission number 14-08) and the ToMMo Ethics Committee (permission numbers 2017-4-26, 2017-4-58, and 2017-4-090). Sequence variations in the *DPYD* gene determined by WGS were confirmed using the polymerase chain reaction (PCR) and Sanger sequencing, according to previously described methods (Akai et al., 2015; Hiratsuka et al., 2015; Hishinuma et al., 2018). The primer pairs used to amplify the *DPYD* gene and Sanger sequencing results containing SNV are listed in Table 3.

**TABLE 3:** Polymerase chain reaction primers used to amplify sequences of the human *DPYD* gene used in this study.

Exon	Primer (5′–3′)		Product length (bp)
	Sense	Antisense	
1	GCTGTCACTTGGCTCTCT	CACCTACCCGCAGAGCA	183
2	GTG​ACA​AAT​GAG​AGA​GAC​CGT​GTC	GCC​TTA​CAA​TGT​GTG​GAG​TGA​GG	285
3	GAA​TGC​TAC​CCA​ATT​AAA​GTG​G	CCT​ACC​ACC​ATC​CTG​TGA​CTG	269
4	GGT​AGA​AAA​TAG​ATT​ATC​TCA​CT	GAA​TTT​ACC​TTG​TTT​GCA​ATA​CT	158
5	GTT​TGT​CGT​AAT​TTG​GCT​G	ATTTGTGCATGGTGATGG	287
6	GAG​GAT​GTA​AGC​TAG​TTT​C	CCA​TTT​GTG​TGC​GTG​AAG​TTC	350
7	GTC​CTC​ATG​CAT​ATC​TTG​TGT​G	GCTTCTGCCTGATGTAGC	361
8	CCT​TAA​TAG​AAC​ATG​TTC​CTG​T	GCA​GTC​ATT​CTG​GAT​ATT​GCT	368
9	AGC​CCC​TCC​TCC​TGC​TAA​T	TGC​TGC​TGA​GCT​TGA​TTT​TG	300
10	GAT​AGT​GAC​ACT​TCA​TCC​TGG	CTGTTGGTGTACAACTCC	340
11	TGG​TGA​AAG​AAA​AAG​CTG​CAT	AAC​AGA​CAA​TTG​CAT​CAC​ACA	347
12	CAG​TTG​TTT​GAA​TCC​CTG​GAA	CGC​CTG​GCC​CAA​TTT​TTA​AT	504
13	CGG​ATG​ACT​GTG​TTG​AAG​TG	TGT​GTA​ATG​ATA​GGT​CGT​GTC	434
14	TGC​AAA​TAT​GTG​AGG​AGG​GAC​C	CAG​CAA​AGC​AAC​TGG​CAG​ATT​C	409
15	CCC​AAA​TGT​CAT​CCA​GTG​T	TTT​CTC​ATG​GCA​GCT​CTT​TAT​TT	335
16	AAC​GGT​GAA​AGC​CTA​TTG​G	TAG​TAA​CTA​TCC​ATA​CGG​GGG	223
17	CAC​GTC​TCC​AGC​TTT​GCT​GTT​G	CGG​GCA​ACT​GAT​TCA​AGT​CAA​G	238
18	TGA​ATG​GGT​TTT​AAC​TAT​CGT​GTC	AAG​TGG​GCA​ACA​CCT​ACC​AG	220
19	TGT​CCA​GTG​ACG​CTG​TCA​TCA​C	CAT​TGC​ATT​TGT​GAG​ATG​GAG	300
20	GAG​AAG​TGA​ATT​TGT​TTG​GAG	CAC​AGA​CCC​ATC​ATA​TGG​CTG	399
21	CGG​AAC​CTG​ATA​CCG​AGA​AG	GCA​GTT​TTC​ACC​ATG​GAC​AG	476
22	GAG​CTT​GCT​AAG​TAA​TTC​AGT​GGC	AGAGCAATATGTGGCACC	288
23	GGG​GAC​AAT​GAT​GAC​CTA​TGT​GG	GGT​GAC​ATG​AAA​GTT​CAC​AGC​AAC	269

### Construction of Expression Vectors

Plasmids containing wild-type human *DPYD* cDNA were constructed as previously described (Hishinuma et al., 2018). Constructs for 43 *DPYD* variants were generated using a QuikChange Lightning Site-Directed Mutagenesis Kit (Agilent Technologies, Santa Clara, CA, United States) according to the manufacturer’s instructions. DPYD*2A was deleted for exon 14 by inverse PCR using the KOD Plus Mutagenesis Kit (TOYOBO, Osaka, Japan). Wild-type and mutant *DPYD* cDNA were then inserted into pcDNA3.4 (Thermo Fisher Scientific, Waltham, MA, United States).

### Dihydropyrimidine Dehydrogenase Variant Expression in 293FT Cells

Human embryonic kidney cell-derived 293FT cells were plated at a density of 2.0 × 10^6^ cells/100-mm dish and transfected with plasmids carrying *DPYD* cDNA (7 μg each) 24 h later using polyethylenimine MAX reagent (Polysciences Inc., Warrington, PA, United States). Sodium sulfide (50 μM), ammonium ferric citrate (50 μM), flavin adenine dinucleotide (50 μM), and flavin mono nucleotide (50 μM) were added 18 h post-transfection. Following incubation for 30 h at 37°C, the cells were scraped off, centrifuged at 1,500 × *g* for 5 min, and resuspended in a homogenization buffer containing 10 mM Tris-HCl (pH 7.4), 1 mM ethylenediaminetetraacetic acid (EDTA), and 10% glycerol. Soluble fractions containing the DPD protein were prepared as previously described (Hishinuma et al., 2018).

### Determination of Dihydropyrimidine Dehydrogenase Protein Expression Using Sodium Dodecyl Sulfate- Polyacrylamide Gel Electrophoresis and Immunoblotting

Proteins were separated by Sodium Dodecyl Sulfate (SDS)-Polyacrylamide Gel Electrophoresis (PAGE), then immunoblotting was performed as previously described (Hishinuma et al., 2018). Protein concentrations were quantified using the bicinchoninic acid assay, and 10 µg of soluble fraction per lane was applied. DPD was detected using a polyclonal anti-human DPD antibody (1:2,000) and goat anti-rabbit IgG (1:5,000). GAPDH antibody was used as a loading control (1:5,000), and goat anti-rabbit IgG (1:10,000) was used as the secondary antibody. Immunoblots were visualized using the SuperSignal West Dura Extended Duration Substrate (Thermo Fisher Scientific). Chemiluminescence was quantified using a ChemiDoc XRS^+^ with Image Lab Software (Bio-Rad Laboratories, Hercules, CA, United States).

### Immunoblotting After Blue Native Polyacrylamide Gel Electrophoresis

Following blue native PAGE, immunoblotting was performed as previously described (Hishinuma et al., 2017; Hishinuma et al., 2018). DPD proteins were detected using a polyclonal anti-human DPD antibody (1:2,000) and goat anti-rabbit IgG (1:5,000). Immunoblots were visualized using the SuperSignal West Femto Maximum Sensitivity Substrate (Thermo Fisher Scientific). NativeMark^TM^ Unstained Protein Standard (Thermo Fisher Scientific) was used as a molecular weight marker, and the proteins were visualized using Coomassie Brilliant Blue (CBB-R250) staining after electrophoresis.

### 5-FU Reduction Assays

5-FU reduction assay was performed as previously described, with several modifications (Hishinuma et al., 2018). 5-FU (0.1–20 μM) was added to the DPD variant protein (20 μg) and incubated at 37°C for 10 min. The reactions were terminated by adding acetonitrile containing 2 μM cytosine-2,4-^13^C_2_,^15^N_3_ as an internal standard.

After removal of proteins by centrifugation, the supernatant was vacuum dried at 40°C for 1.5 h and redissolved in 0.1% (v/v) formic acid solution. Sample solutions (5 μl) were injected into a high-performance liquid chromatography-tandem mass spectrometry (HPLC-MS/MS) system, and FUH_2_ was measured using the positive ion detection mode. A standard curve was prepared using FUH_2_ standards in the range of 0.01–10 μM. Enzyme activity was normalized using the corresponding DPD expression levels quantified by immunoblotting after SDS-PAGE.

### Data Analysis

Kinetic data were analyzed using SigmaPlot 12.5 Enzyme Kinetics Module (Systat Software Inc., Chicago, IL, United States); Michaelis constant (*K*_*m*_), maximum velocity (*V*_*max*_), and intrinsic clearance (*CL*_*int*_ = *V*_*max*_/*K*_*m*_) values were determined using the average DPD expression values obtained in triplicate and are expressed as mean ± standard deviation. Statistical variance in protein expression and kinetic parameters was analyzed using Dunnett’s t-test, Dunnett’s T3 test, or the Kruskal-Wallis method (IBM SPSS Statistics Ver. 22, International Business Machines, Armonk, NY, United States). Statistical significance was set at *p* < 0.05.

### 3D Simulation Modeling of Dihydropyrimidine Dehydrogenase

A 3D simulation modeling analysis of DPD was performed as previously described (Hishinuma et al., 2018). Discovery Studio 4.5 was used for human DPD 3D imaging, and the Discovery Studio 2.5 (Accelrys, San Diego, CA, United States) CDOKER protocol was used to create docked DPD–5-FU structures.

## Results

### Confirmation of *DPYD* Exonic Single Nucleotide Variants Identified in 3,554 Japanese Individuals

A total of 41 *DPYD* allelic variants, including 15 novel variants in Japanese participants, were previously detecting using WGS (Tadaka et al., 2021). To confirm these alterations, Sanger sequencing was performed on *DPYD* variants according to previously described methods (Akai et al., 2015; Hiratsuka et al., 2015; Hishinuma et al., 2018). All exonic SNV and WGS results matched those obtained by Sanger sequencing (Table 1).

### Wild-Type and Dihydropyrimidine Dehydrogenase Variant Expression Levels in 293FT Cells

**FIGURE 2:**
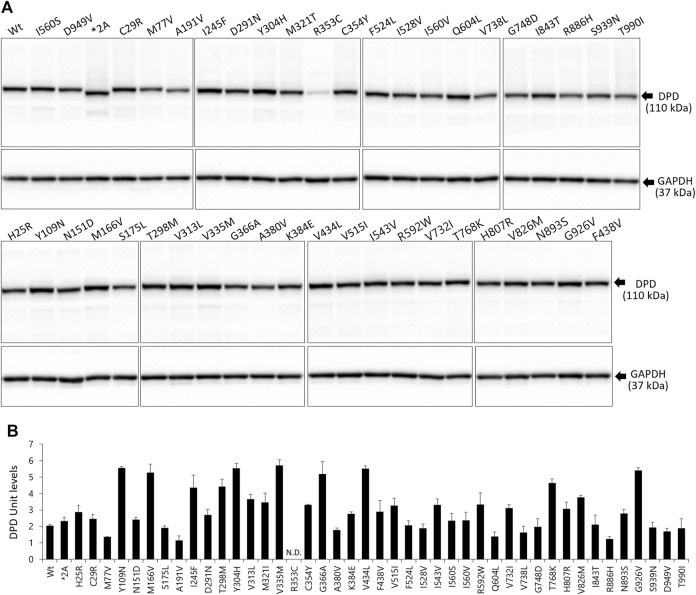
Expression levels of wild-type and variant DPD proteins. **(A)** DPD protein levels were determined by immunoblotting analysis following SDS-PAGE. **(B)** DPD units of proteins expressed in 293FT cells. Each bar represents the mean ± standard deviation of three independent assays.

DPD expression levels were determined by immunoblotting after SDS-PAGE with a polyclonal anti-human DPD antibody that recognizes all DPD variants (Figure 2A). The band for the DPYD*2A variant was detected at a lower molecular weight owing to skipping of exon 14. GAPDH expression levels were constant in all transfected 293FT cells. Endogenous DPD protein was not detected in the soluble fraction of 293FT cells.

The average levels of the wild-type and variant DPD proteins are shown in Figure 2B. We defined DPD expression levels equivalent to 10 μg of wild-type DPD as 1 DPD unit. The R353C variant was below the lower limit of quantification; therefore, the expression levels could not be determined. There were no significant differences in the expression levels of other variants, except for the R353C variant, compared to the wild-type.

### Kinetics of 5-FU Reduction Mediated by Dihydropyrimidine Dehydrogenase

**FIGURE 3:**
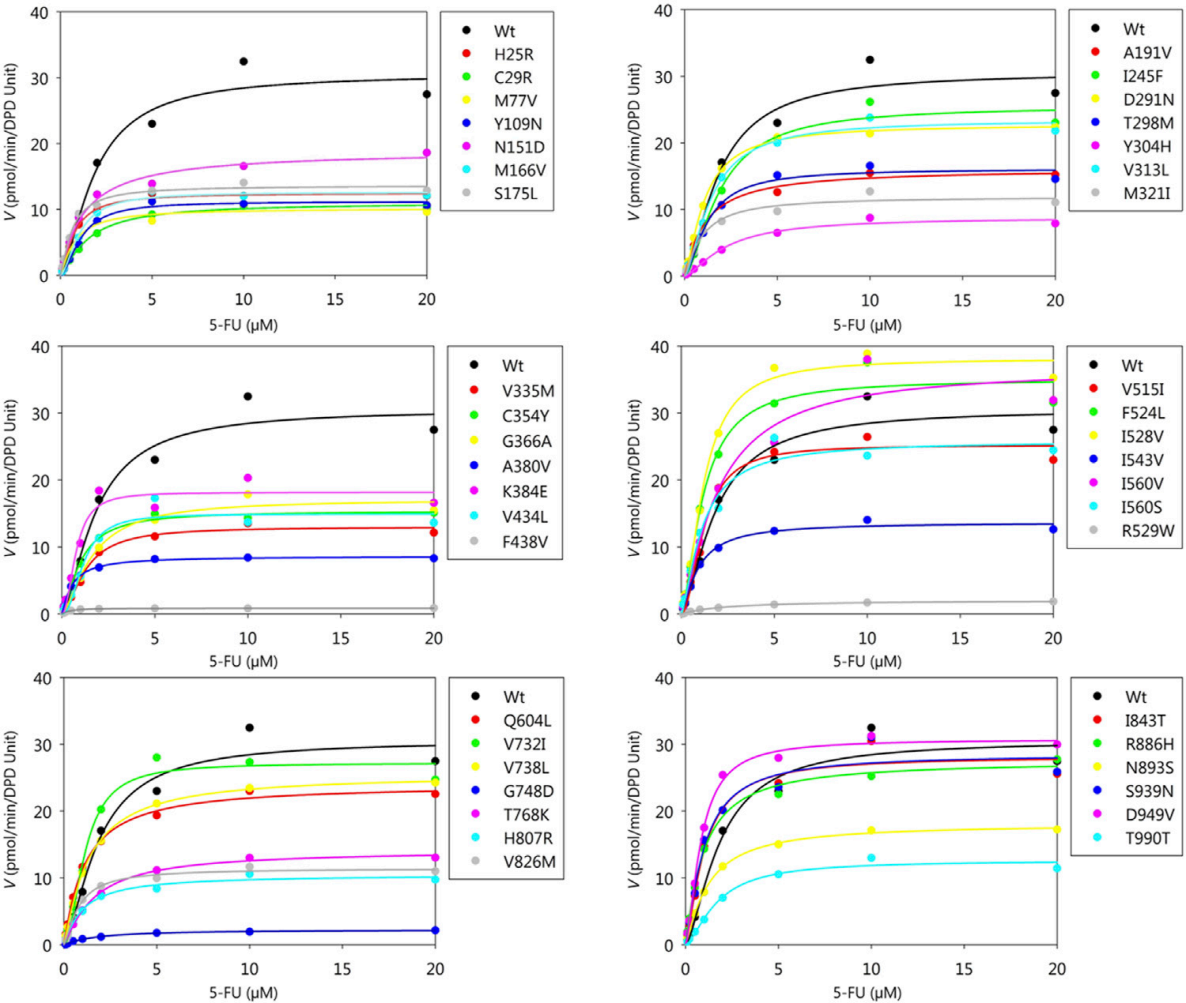
Michaelis-Menten curves of DPD variants. The kinetic parameters *K*_*m*_, *V*_*max*_, and *CL*_*int*_ of 5-FU reduction were determined. The velocities for the reduction of 5-FU and 5-FU concentration are plotted on the horizontal and vertical axes, respectively.

Kinetic parameters of 5-FU reduction were calculated for the wild-type and 44 DPD variants (Table 4). The nonlinear regression curves of the Michaelis-Menten equation are shown in Figure 3. Kinetic parameters could not be determined for R353C, DPYD*2A, and G926V, as no 5-FU reduction activity was detected.

**TABLE 4:** Kinetic parameters of dihydropyrimidine dehydrogenase (DPD) variants in 5-FU metabolism.

Variants	*K*_*m*_ (μM)	*V*_*max*_ (pmol/minute/DPD unit)	*CL*_*int*_ (*V*_*max*_ */K*_*m*_) (μl/minute/DPD unit)	% of Wild-type *CL*_*int*_
Wild-type	1.60 ± 0.10	31.56 ± 1.00	19.77 ± 0.66	100.00
H25R	0.75 ± 0.04	12.53 ± 0.23	16.82 ± 0.83	85.10
C29R	1.57 ± 0.21	11.15 ± 0.66	7.14 ± 0.53	36.14
M77V	0.77 ± 0.06	10.15 ± 0.47	13.20 ± 0.85	66.79
Y109N	1.12 ± 0.13	11.19 ± 0.33	10.11 ± 1.11	51.17
N151D	2.81 ± 3.11	21.73 ± 7.27	13.25 ± 7.69	67.03
M166V	1.03 ± 0.09	12.62 ± 1.21	12.20 ± 0.18	61.74
S175L	0.62 ± 0.04	13.61 ± 0.70	21.88 ± 1.06	110.66
A191V	1.11 ± 0.04	16.10 ± 1.06	14.49 ± 0.94	73.32
I245F	1.94 ± 0.19	25.70 ± 1.27	13.36 ± 1.36	67.57
D291N	1.08 ± 0.22	22.77 ± 2.33	21.35 ± 2.60	108.00
T298M	1.23 ± 0.14	16.12 ± 0.81	13.16 ± 0.81	66.58
Y304H	2.22 ± 0.24	8.79 ± 0.84	3.97 ± 0.12	20.07
V313L	1.46 ± 0.30	23.46 ± 2.86	16.35 ± 1.80	82.73
M321I	0.96 ± 0.15	11.99 ± 0.54	12.62 ± 1.55	63.83
V335M	1.27 ± 0.01	13.04 ± 0.47	10.25 ± 0.46	51.84
C354Y	0.99 ± 0.08	15.35 ± 0.34	15.61 ± 0.95	78.96
G366A	1.62 ± 0.18	17.11 ± 1.64	10.61 ± 0.40	53.66
A380V	0.56 ± 0.04	8.62 ± 0.39	15.48 ± 0.52	78.30
K384E	0.80 ± 0.10	18.49 ± 0.65	23.29 ± 2.50	117.80
V434L	1.15 ± 0.04	14.94 ± 0.99	12.94 ± 0.50	65.45
F438V	0.40 ± 0.01	0.86 ± 0.04	2.15 ± 0.17	10.87
V515I	1.22 ± 0.08	25.31 ± 1.67	20.81 ± 0.14	105.28
F524L	1.18 ± 0.09	35.15 ± 1.15	29.80 ± 1.73	150.77
I528V	1.22 ± 0.18	38.44 ± 2.65	31.78 ± 2.86	160.76
I543V	0.99 ± 0.35	13.89 ± 2.11	14.71 ± 2.60	74.41
I560V	1.98 ± 0.13	36.86 ± 1.30	18.63 ± 0.72	94.22
I560S	1.28 ± 0.43	26.68 ± 4.42	21.70 ± 4.37	109.75
R592W	2.49 ± 0.28	2.09 ± 0.08	0.84 ± 0.08	4.27
Q604L	1.12 ± 0.11	23.99 ± 0.34	21.50 ± 1.82	108.74
V732I	1.14 ± 0.06	27.36 ± 1.82	23.93 ± 1.22	121.06
V738L	1.36 ± 0.19	25.67 ± 1.51	19.04 ± 1.52	96.30
G748D	2.25 ± 1.31	2.38 ± 0.27	1.24 ± 0.45	6.26
T768K	1.69 ± 0.42	14.22 ± 1.56	8.62 ± 1.23	43.58
H807R	1.06 ± 0.26	10.60 ± 1.56	10.19 ± 0.92	51.54
V826M	0.83 ± 0.14	11.46 ± 0.61	14.05 ± 1.60	71.08
I843T	1.00 ± 0.11	28.28 ± 1.63	28.25 ± 1.58	142.92
R886H	0.96 ± 0.09	27.57 ± 0.72	28.82 ± 1.99	145.81
N893S	1.27 ± 0.13	18.27 ± 0.54	14.46 ± 1.33	73.15
S939N	1.00 ± 0.22	28.68 ± 3.09	29.38 ± 3.93	148.64
D949V	0.85 ± 0.08	30.99 ± 0.37	36.77 ± 3.19	186.01
T990I	1.77 ± 0.15	12.81 ± 0.55	7.29 ± 0.89	36.86

Data represent the mean ± standard deviation of three independent catalytic assays. The kinetic parameters of R353C, DPYD*2A, and G926V could not be determined because enzymatic activity was not detected at the highest substrate concentration used in the assay (200 μM 5-FU). *K*_*m*_, Michaelis constant; *V*_*max*_, maximum velocity; *CL*_*int*_, intrinsic clearance.

The *K*_*m*_, *V*_*max*_, and *CL*_*int*_ values for the reduction of 5-FU by wild-type DPD were 1.60 μM, 31.56 pmol/min/DPD unit, and 19.77 μl/min/DPD unit, respectively. None of the parameters showed significant differences because they did not follow a normal distribution; therefore, nonparametric tests were performed, but seven DPD variants (C29R, Y304H, F438V, R592W, G748D, T768K, and T990I) showed 50% or lower decrease in *CL*_*int*_ compared to the wild-type. On the other hand, three variants (F524L, I528V, and D949V) showed 150% or higher increase in *CL*_*int*_ compared to the wild-type.

### Blue Native Polyacrylamide Gel Electrophoresis Analysis of Dihydropyrimidine Dehydrogenase Variants

**FIGURE 4:**
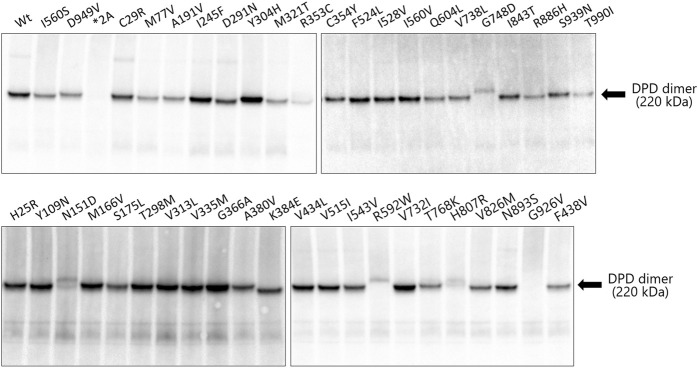
Immunoblotting analysis after blue native PAGE showing immunoreactive dihydropyrimidine dehydrogenase (DPD) variant proteins. Blue native PAGE was performed using Tris-glycine buffer and 5%–20% polyacrylamide gels; 9 μg of the soluble fraction of DPD variant proteins were loaded into each lane in triplicate. DPD variants were detected using polyclonal antibodies against human DPD.

Immunoblotting results after blue native PAGE are shown in Figure 4. The molecular weight of 43 variants, including wild-type DPD but excluding the DPYD*2A and G926V variants that lost enzymatic activity, was around 242 kDa, which corresponds to the DPD dimer. Six DPD variants (N151D, R353C, R592W, G748D, H807R, and T990I) showed a decreased band intensity compared to that of other variants. In addition, electrophoretic mobility was reduced in four variants (N151D, R592W, G748D, and H807R).

### 3D Structural Modeling of Dihydropyrimidine Dehydrogenase

**FIGURE 5:**
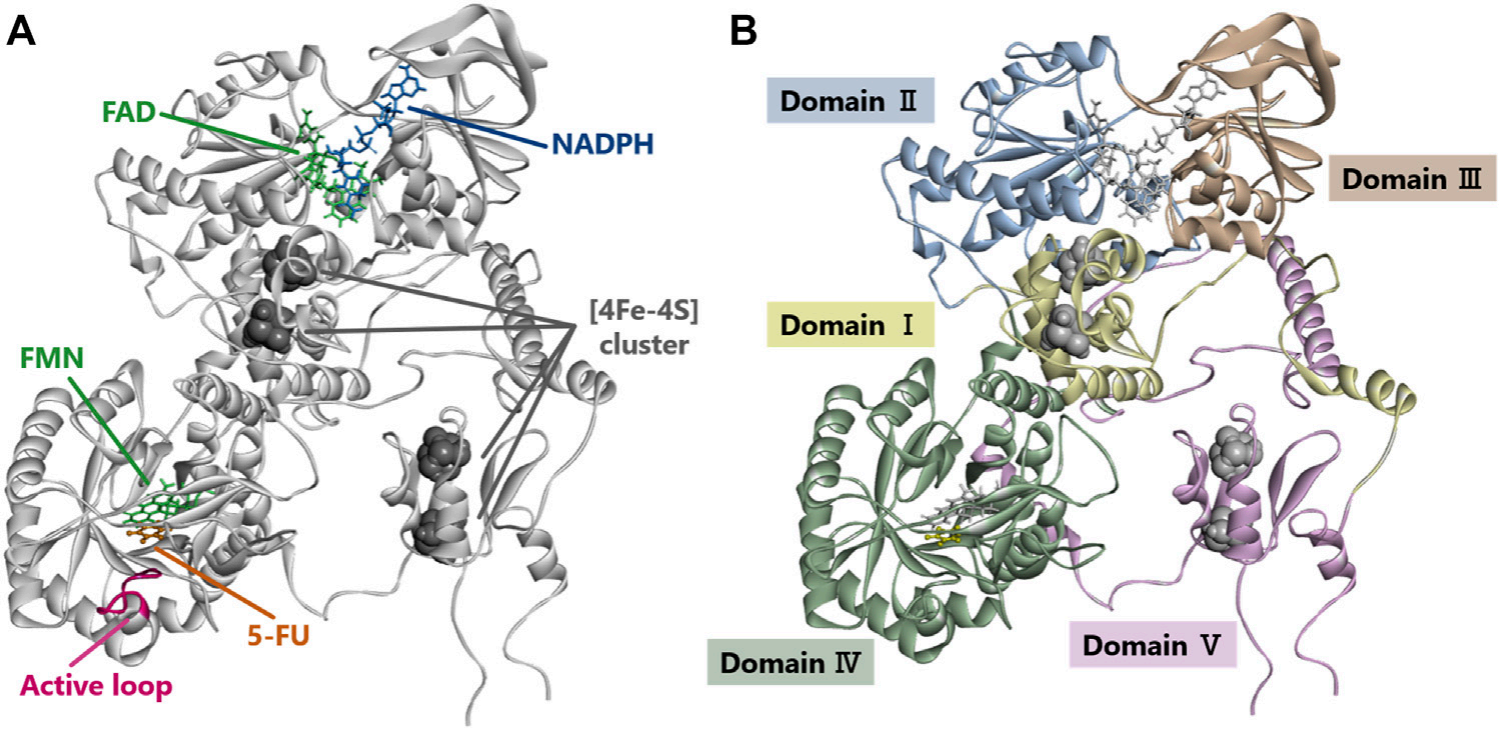
Dihydropyrimidine dehydrogenase (DPD) structural analysis. **(A)** Diagram showing overall structure of human DPD. **(B)** Diagram showing each domain of human DPD.

**FIGURE 6:**
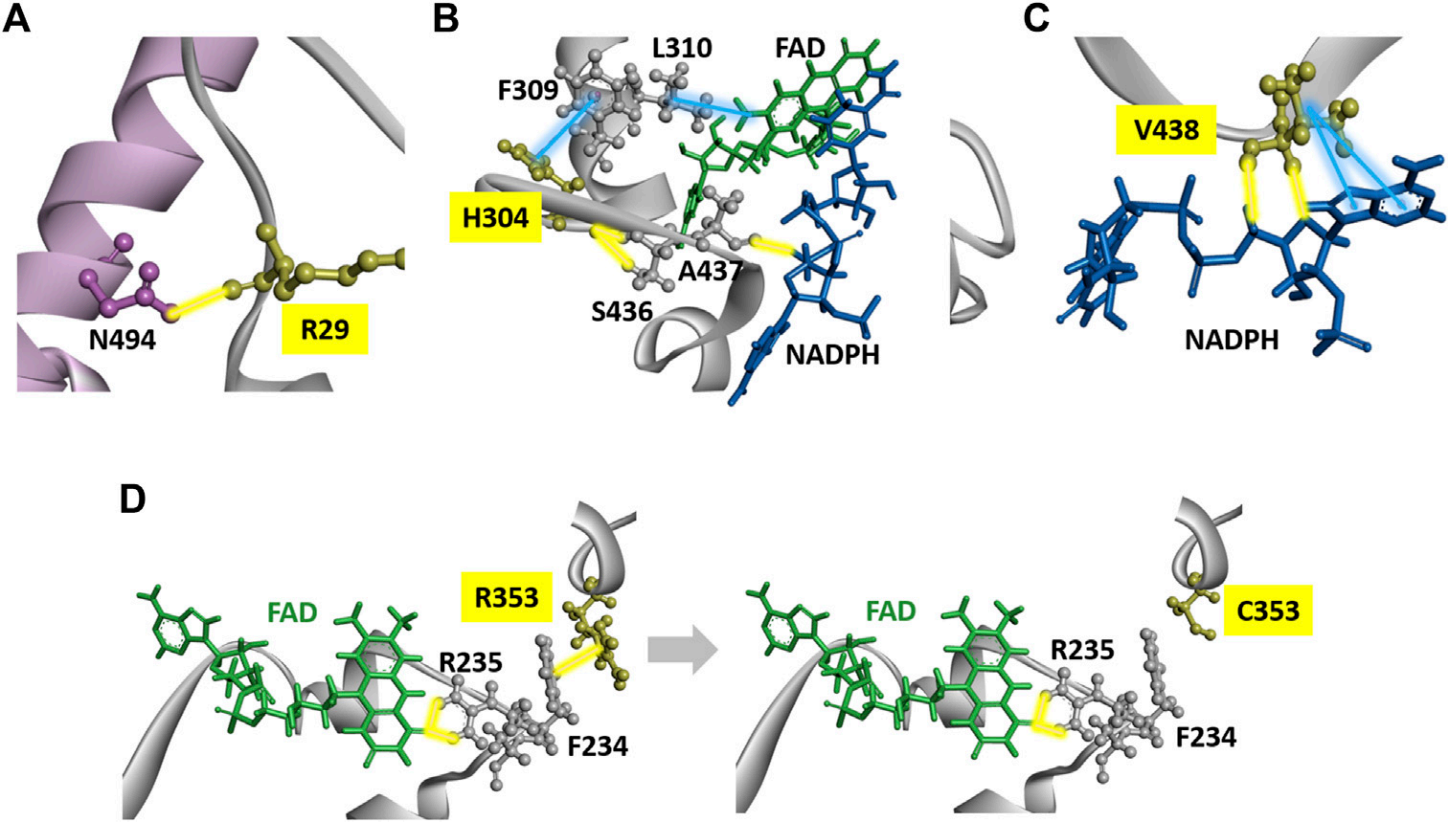
DPD structural analysis. **(A)** Diagram of a fragment of the crystal structures of the H25R variant. The R25 residues are shown in yellow. **(B)** Diagram of a fragment of the crystal structures of the H304Y variant. The Y304 residues are shown in yellow. **(C)** Diagram of a fragment of the crystal structures of the F438V variant. The F438 residues are shown in yellow. **(D)** Diagrams of a fragment of the crystal structures of wild-type DPD (left panel) and R353C (right panel). The R353 and C353 residues are shown in yellow. Yellow and blue lines indicate hydrogen bonds and hydrophobic interactions, respectively.

The crystal structure and five domains of human DPD created by homology modeling are shown in Figures 5A,B, respectively. Close-up views of the 3D crystal structure models of the C29R, Y304H, and F438V mutation sites are shown in Figures 6A–C, respectively. The R29 residue formed a hydrogen bond with N494. The H304 residue was bound to FAD via hydrophobic interactions with F309 and L310, and to NADPH *via* hydrogen bonds with S436 and A437. The V438 residue formed a direct hydrogen bond and hydrophobic interaction with NADPH. A close-up view of the R353C mutation site is shown in Figure 6D. The R353 residue was bound to FAD via hydrogen bonds with F234 and R235; however, substitution with C353 eliminated these interactions.

## Discussion

DPD deficiencies can lead to the development of severe 5-FU toxicity; however, significant geographical-ethnicity differences exist with regards to polymorphisms of the *DPYD* gene (Table 2) (Lunenburg et al., 2016; Roberto et al., 2017). Therefore, it is important to identify genomic biomarkers for each ethnic group. In this study, we used *in vitro* assays to evaluate changes in the enzymatic function of DPD variants derived from 41 *DPYD* genetic polymorphisms identified by WGS in a Japanese population of 3,554 individuals along with three risk variants previously reported as predictive markers of 5-FU toxicity in Caucasians (Amstutz et al., 2018; Lunenburg et al., 2020). We previously demonstrated that the addition of four cofactors (iron, sulfur, FAD, and FMN) in the *in vitro* expression system of the DPD protein is crucial for the functional analysis of DPD variants (Hishinuma et al., 2018). Therefore, we transiently expressed 44 DPD variants in 293FT cells in the presence of these four cofactors, and evaluated the enzymatic activity of each DPD variant by determining the kinetic parameters of 5-FU reduction. The kinetic parameters of three variants (R353C, DPYD*2A, and G926V) could not be determined because no enzymatic activity was detected at the highest substrate concentration used in the assay. In addition, seven DPD variants (C29R, Y304H, F438V, R592W, G748D, T768K, and T990I) showed a *CL*_*int*_ less than 50% of the wild-type DPD. DPYD*2A, which has been reported as a predictive marker of 5-FU toxicity in Caucasians, is known to lose enzymatic activity due to skipping of exon 14 (Van Kuilenburg et al., 1997; Nie et al., 2017). Consistent with our results, Offer et al. (2013) reported that recombinant DPD expressed in HEK293T/c17 cells also lost enzymatic activity. In contrast, two risk variants (I560S and D949V) showed increased enzymatic activity in the *in vitro* analysis of this study. *In vivo* and *in vitro* differences between these variants may be due to differences in expression conditions and cell lines, and may also be due to differential regulation of their expression by mRNA or other factors *in vivo*.

The DPD molecule is composed of five domains: domains I and V contain two (4Fe-4S) clusters of two molecules each, domain II contains the FAD binding site, and domain IV contains the FMN binding site. NADPH, which is required for reduction, binds to domain III to provide electrons, and the active site is located in domain IV (Lu et al., 1992; Dobritzsch et al., 2001; Schnackerz et al., 2004; Lohkamp et al., 2010). The amino acid sequences of these domains are highly conserved among various animal species, and the ability to form dimers plays an important role in the activity of DPD since it is activated by dimerization and the transporting of electrons through the (4Fe-4S) clusters (Dobritzsch et al., 2002; Lohkamp et al., 2010). To determine the effect of amino acid substitutions on DPD dimer formation we performed immunoblotting after blue native PAGE under non-denaturing conditions. A band of approximately 242 kDa, estimated to be a dimer, was detected in all DPD variants except DPYD*2A and G926V, for which a loss of enzymatic activity was observed. In addition, the resulting band of seven DPD variants (N151D, R353C, R592W, G748D, T768K, H807R, and T990I) had a decreased intensity compared to that of other variants, and these variants showed either decreased or no enzymatic activity. Furthermore, four variants (N151D, R592W, G748D, and H807R) showed a decrease in electrophoretic mobility that could be attributed to a change in the charge of the amino acids. These results suggest that these variants have reduced activity due to amino acid substitutions and changes in the charge of amino acids that prevent the dimerization of DPD. Three variants (R592W, T768K, and G926V) showed reduced enzymatic activity, similar to our previously reported analysis of DPD variants identified in 1,070 Japanese individuals (Hishinuma et al., 2018). In contrast, the C29R, Y304H, and F438V variants had a *CL*_*int*_ less than 50% of the wild-type, although the intensity of their bands resulting from blue native PAGE were the same intensity as that of wild-type, and there was no clear correlation between the *CL*_*int*_ value and dimerization ability in 5-FU reduction activity. It is possible that amino acid substitutions in these variants affected domain functions other than dimer formation.

The C29R variant has an allele frequency of 3.05% in the Japanese population, and is a common variant worldwide (Maekawa et al., 2007; Caudle et al., 2013). In this study, the C29R variant reduced the *CL*_*int*_ of 5-FU reduction activity to 36% of wild-type DPD. Although the C29R variant did not show any alteration in side-chain binding or interaction in the 3D simulation analysis, this change occurs in domain I, and the substitution from cysteine to arginine might affect the transportation of electrons. Offer et al. (2013) reported that the C29R variant showed 13% higher activity than wild-type when expressed in HEK293T/c17 cells. In contrast, Kuilenburg et al. reported that the enzymatic activity of the C29R variant was reduced to approximately 80% of that of wild-type when expressed in HEK293 Flp-In cells (Kuilenburg et al., 2016). While there are reports of an increased incidence of grade 3–4 diarrhea in response to 5-FU treatment in patients carrying the C29R variant, there are also reports of no 5-FU toxicity developing in these individuals, and the relevance of this variant to the risk of developing 5-FU toxicity remains unclear (Joerger et al., 2015; Khushman et al., 2018; Maharjan et al., 2019).

In our study, the Y304H variant reduced the *CL*_*int*_ of 5-FU reduction activity to 20% of that of wild-type DPD. Offer et al., 2013 reported that the enzymatic activity of the Y304H variant was reduced to approximately 80% of that of wild-type DPD in HEK293T/c17 cells (Offer et al., 2014). Y304 is in domain III and forms hydrophobic interactions or hydrogen bonds with F309 near FAD and S436 near NADPH. The F438V variant reduced the *CL*_*int*_ of 5-FU reduction activity to 11% of that of wild-type DPD. F438 is also present in domain III and forms hydrophobic interactions and hydrogen bonds with NADPH. Although no changes in side chain binding or interactions were observed in the 3D simulation analysis of either variant, domain III binds NADPH, an important source of electrons, and it is assumed that the amino acid substitution affected the binding of NADPH, resulting in a decrease in enzymatic activity.

Offer et al., 2013 reported no enzymatic activity of the R353C variant when expressed in HEK293T/c17 cells (Offer et al., 2014). In the present study, the R353C variant also lost enzymatic activity. R353 is in domain III, and its electrostatic interaction with F234 was abolished by the substitution to C353. Since R235, located next to F234, forms a hydrogen bond with FAD, these results suggest that the substitution to C353 caused the loss of enzymatic activity due to a conformational change around FAD.

In conclusion, the wild-type and 41 DPD variants identified in a Japanese population of 3,554 individuals were expressed in 293FT cells, and their enzymatic activities were assessed *in vitro*. The results showed that nine DPD variants (C29R, Y304H, R353C, F438V, R592W, G748D, T768K, G926V, and T990I) reduced enzymatic activity to less than 50% of that of wild-type DPD. Therefore, we hypothesized that patients with these *DPYD* polymorphisms may develop severe toxicity due to elevated concentrations of 5-FU during chemotherapy. Except for C29R, these variants have been identified at very low frequencies and mostly in Asian populations, suggesting that several rare *DPYD* polymorphisms causing reduced DPD activity may be responsible for potential 5-FU toxicity in populations where the four risk variants have not been identified. Therefore, we suggest that rare *DPYD* polymorphism may be a useful predictive marker for 5-FU toxicity in the Japanese population. However, this information is insufficient to determine whether these variants affect the pharmacokinetics and pharmacodynamics of 5-FU *in vivo*. It is expected that large-scale clinical trials will be conducted in the future to analyze the correlation between the *DPYD* genotype and the blood levels of 5-FU and its derivatives in patients to evaluate its usefulness as a genetic biomarker for predicting 5-FU toxicity.

## Data Availability

The datasets presented in this study can be found in online repositories. The names of the repository/repositories and accession number(s) can be found in the article/supplementary material.
